# Genetic variants in *ARID5B* and *CEBPE* are childhood ALL susceptibility loci in Hispanics

**DOI:** 10.1007/s10552-013-0256-3

**Published:** 2013-07-09

**Authors:** Anand P. Chokkalingam, Ling-I Hsu, Catherine Metayer, Helen M. Hansen, Stacy R. Month, Lisa F. Barcellos, Joseph L. Wiemels, Patricia A. Buffler

**Affiliations:** 1 Division of Epidemiology, School of Public Health, University of California, 1995 University Ave, Ste 460, Berkeley, CA 94704 USA; 2 Department of Epidemiology and Biostatistics, School of Medicine, University of California, San Francisco, CA USA; 3 Kaiser Permanente, Oakland, CA USA

**Keywords:** Childhood cancer, Leukemia, Genetic susceptibility

## Abstract

Recent genome-wide studies conducted in European Whites have identified novel susceptibility genes for childhood acute lymphoblastic leukemia (ALL). We sought to examine whether these loci are susceptibility genes among Hispanics, whose reported incidence of childhood ALL is the highest of all ethnic groups in California, and whether their effects differ between Hispanics and non-Hispanic Whites (NHWs). We genotyped 13 variants in these genes among 706 Hispanic (300 cases, 406 controls) and 594 NHW (225 cases, 369 controls) participants in a matched population-based case–control study in California. We found significant associations for the five studied *ARID5B* variants in both Hispanics (*p* values of 1.0 × 10^−9^ to 0.004) and NHWs (*p* values of 2.2 × 10^−6^ to 0.018). Risk estimates were in the same direction in both groups (ORs of 1.53–1.99 and 1.37–1.84, respectively) and strengthened when restricted to B-cell precursor high-hyperdiploid ALL (>50 chromosomes; ORs of 2.21–3.22 and 1.67–2.71, respectively). Similar results were observed for the single *CEBPE* variant. Hispanics and NHWs exhibited different susceptibility loci at *CDKN2A*. Although *IKZF1* loci showed significant susceptibility effects among NHWs (*p* < 1 × 10^−5^), their effects among Hispanics were in the same direction but nonsignificant, despite similar minor allele frequencies. Future studies should examine whether the observed effects vary by environmental, immunological, or lifestyle factors.

## Introduction

Acute lymphoblastic leukemia (ALL) is the most common cancer among children under the age of 15 in Western countries, and its etiology is poorly understood. In 2009, two genome-wide genetic susceptibility studies based on European White populations identified novel loci for the risk of childhood ALL in *ARID5B* (10q21.2), *IKZF1* (7p12.2), and *CEBPE* (14q11.2) [1, 2]. In both studies, the loci in *ARID5B* were found to have particularly strong effects for the B-cell precursor hyperdiploid subtype of childhood ALL [1, 2]. These associations have been confirmed in additional European White populations [3–5]. Variation in *ARID5B* (rs10821936) has been associated with ALL risk in African American, Thai, and Hispanic populations [6–8]. Furthermore, a locus in *CDKN2A* (9p21.3) that did not reach genome-wide significance in one of the original genome-wide studies [2] was found to be significantly associated with the risk of childhood ALL using a larger European White validation set [9].

Most genome-wide studies on childhood ALL conducted to date have been limited to White populations. Understanding the effects of these variants in different populations is crucial because reported incidence rates of childhood ALL among California’s population who self-identify as Hispanic ethnicity (“Hispanics”) are higher than those for any other racial/ethnic subgroup in California, including non-Hispanic Whites [10]. Reasons for these higher rates are unclear. In addition, Hispanics tend to have a worse prognosis versus non-Hispanic Whites, and *ARID5B* has been linked to poorer outcomes [8]. Hispanics are a recently genetically admixed group [11], and we have previously observed differences in haplotype structure between Hispanics and non-Hispanics [12], suggesting that they may not share the same susceptibility loci. Therefore, it is possible that the higher observed ALL incidence rates among Hispanics may be related to differences in genetic susceptibility loci, including those in *ARID5B*, *IKZF1*, *CEBPE*, and *CDKN2A*.

The current study based in California examines the role of these loci in childhood ALL risk among Hispanics and ascertains whether the effects of these loci differ between self-reported Hispanics and non-Hispanic Whites.

## Materials and methods

### Study subjects

The study was conducted among participants in the Northern California Childhood Leukemia Study (NCCLS), whose recruitment and enrollment procedures have been described in detail previously [13]. Briefly, this population-based case–control study started in 1995 and recruited subjects from 35 counties in Northern and Central California. Case subjects newly diagnosed with leukemia were recruited from nine hospitals in the catchment area, usually within 72 h of diagnosis. Comparison with the California Cancer Registry (1997–2003) showed that the NCCLS case ascertainment protocol has captured ~95 % of children diagnosed with leukemia in the participating study hospitals. When considering both participating and nonparticipating hospitals within the study region, cases ascertained through the NCCLS represent ~76 % of all diagnosed cases. Birth certificate information obtained from the Office of Vital Records at the California Department of Public Health (CDPH) was used to select one to two controls for each case, matching on date of birth, sex, Hispanic ethnicity (at least one parent self-reporting Hispanic ethnicity), and maternal race (White, Black, Asian/Pacific Islander, Native American, and Other/Mixed).

The child’s own race/ethnicity was defined according to that of both parents. For example, a child was considered non-Hispanic White if both parents reported being non-Hispanic ethnicity and White race. Children of parents reporting different races were considered to be of Mixed/Other race. Any child with a parent reporting Hispanic ethnicity was considered Hispanic, regardless of parental race. The eligibility criteria for cases and controls were as follows: (a) residency in the study area, (b) being younger than 15 years at case diagnosis (reference date for the matched controls), (c) having at least one English- or Spanish-speaking parent or guardian, and (d) having not been previously diagnosed with cancer. Interview rates among eligible cases and controls were 80 and 84 %, respectively, and did not vary markedly by Hispanic ethnicity.

The current analysis includes Hispanic and non-Hispanic White ALL case and control subjects recruited between 1995 and 2008 who had available DNA specimens. These two racial/ethnic groups together comprise ~85 % of enrolled subjects. A child was considered Hispanic if either parent self-reported Hispanic ethnicity, regardless of self-reported parental race. Non-Hispanic White subjects were those whose parents both self-reported as being of non-Hispanic ethnicity and White race. Other race/ethnicity groups (non-Hispanic Blacks, non-Hispanic Asians and Pacific Islanders, non-Hispanic Native Americans, and non-Hispanic Mixed) were not considered due to small number of subjects in each. Children less than 1 year of age at diagnosis/reference date were excluded due to growing evidence that these leukemias may be etiologically distinct compared to leukemia diagnosed at later ages [14]. Based on the previous reports of stronger effects for B-cell precursor (BCP) ALL and specifically BCP high-hyperdiploid ALL (>50 chromosomes), we also considered these two subgroups in our analyses. The cytogenetic classification methods used in this analysis have been described in detail elsewhere [15]. Briefly, pretreatment diagnostic karyotype and fluorescence in situ hybridization (FISH) data were abstracted from leukemia patient records shortly after diagnosis. Additional FISH analyses were conducted at the University of California, Berkeley, to identify hyperdiploidy when not done at hospitals.

This study was reviewed and approved by institutional review committees at the University of California Berkeley, the CDPH, and the participating hospitals. Written informed consent was obtained from all parent respondents.

### DNA processing and genotyping

DNA specimens from buccal cytobrushes collected by trained interviewers from 95 % of participating children (cases and controls) were processed within 48 h of collection by heating in the presence of 0.5 N NaOH. DNA thus isolated was later repurified either manually using Gentra Puregene reagents (QIAGEN, USA, Valencia, CA) or an automated organic DNA extraction protocol (AutoGen, Holliston, MA). Whole-genome amplification (WGA) of buccal cell DNA was performed using GenomePlex reagents (Rubicon Genomics, Ann Arbor, MI) according to the manufacturer’s protocol. WGA products were cleaned with a Montage PCR9 filter plate (Millipore, Billerica, MA). When buccal cytobrush DNA was inadequate or not available (26.6 % of subjects), DNA was isolated from dried bloodspots collected at birth and archived by the Genetic Diseases Screening Program of the CDPH. After extraction using the QIAamp DNA Mini Kit (QIAGEN, USA, Valencia, CA), these DNA samples were whole-genome amplified using REPLI-g reagents (QIAGEN, USA, Valencia, CA). We previously genotyped DNA specimens from both buccal cells and DBS for nine subjects; genotype concordance between paired samples was 98.9 % [16]. Regardless of source, DNA specimens were quantified using human-specific Alu-PCR to confirm a minimum level of amplifiable human DNA [17] and randomized prior to genotyping.

We performed Sequenom iPlex genotyping of 11 of 13 previously identified SNPs [1, 2, 9] as follows: *ARID5B* (rs10994982, rs10740055, rs7073837, rs7089424, and rs10821936), *IKZF1* (rs11978267), *CEBPE* (rs2239633), and *CDKN2A* (rs3731217, rs3218018, rs2811712, and rs3731239). The average SNP call rate was 96.7 %. Genotypes for duplicate DNA specimens (*n* = 154 per SNP) showed 100 % concordance. The two remaining SNPs, one in *IKZF1* (rs4132601) and the other in *CDKN2A* (rs4074785), were typed using TaqMan assays. The average SNP call rate was 98.2 %. Genotypes for duplicate DNA specimens (*n* = 146 per SNP) showed 100 % concordance. All SNPs were tested for deviation from Hardy–Weinberg equilibrium using SAS version 9.2 software, stratified by ethnicity. SNPs were excluded from statistical analyses if they had a call rate of <90 %, had a minor allele frequency <5 %, or failed Hardy–Weinberg equilibrium (*p* < 0.01) in both Hispanic and non-Hispanic White controls.

### Statistical methods

Logistic regression was used to estimate the association of individual SNPs with the risk of childhood ALL. Although individual matching was used in the study design, because not all cases had a matched control by the time genotyping was conducted, we used unconditional logistic regression adjusted for the matching factors (age at diagnosis and sex, as well as child’s race for Hispanics only) to ensure data for all available subjects were included. Analyses were conducted for total ALL, for BCP ALL, and for BCP high-hyperdiploid ALL, stratified by ethnicity (non-Hispanic Whites versus Hispanics). We used log-additive inheritance models to test for trend in association with copies of the minor allele. For each SNP, the referent allele was set to match that reported previously [1, 2, 9], even in instances where this allele was less common in our study populations. Results are reported separately for Hispanics and non-Hispanic Whites. *p* values < 0.05 were considered statistically significant.

## Results

A total of 706 Hispanics (300 cases and 406 controls) and 594 non-Hispanic Whites (225 cases and 369 controls) were included in this study (Table 1). As expected, there was some racial variation among Hispanics, with a sizable proportion (59–68 %) reporting “Mixed/Other” race. The frequency of BCP ALL was 93.0 and 89.8 % among the Hispanic and non-Hispanic White cases, respectively, while the prevalence of BCP high-hyperdiploid ALL (>50 chromosomes) was 28.3 and 27.6 %, respectively.

**Table 1:** Characteristics of study population

	Hispanic		Non-Hispanic Whites	
	Cases	Controls	Cases	Controls
Total	300	406	225	369

Age of child	Mean	SD	Mean	SD	Mean	SD	Mean	SD
Years	5.6	3.6	5.5	3.6	5.6	3.4	5.7	3.8

Sex of child	N	%	N	%	N	%	N	%
Male	156	52.0	214	52.7	125	55.6	221	59.9
Female	144	48.0	192	47.3	100	44.4	148	40.1
Race of child								
White	114	38.0	116	28.6	225	100	369	100
African American	2	0.7	0	0.0	–	–	–	–
Native American	6	2.0	11	2.7	–	–	–	–
Asian/Pacific Islander	0	0.0	3	0.7	–	–	–	–
Mixed/other	178	59.3	276	68.0	–	–	–	–
Cytogenetic grouping (cases only)								
Total B-cell precursor ALL	279	93.0	–	–	202	89.8	–	–
B-cell precursor high-hyperdiploid ALL	85	28.3	–	–	62	27.6	–	–

Association tests and ORs for the defined minor alleles of all 13 SNPs are shown in Table 2. Among non-Hispanic Whites, we observed significant associations with the risk of total ALL with all five *ARID5B* variants (*p* values from 0.018 to 2.2 × 10^−6^). Risk estimates for the *ARID5B* variants increased when restricted to BCP high-hyperdiploid ALL; this effect was particularly marked for rs7073837, rs7089424, and rs10821936. The *CEBPE* variant rs2239633 was also significantly associated with total ALL risk among non-Hispanic Whites (*p* = 0.005), and the effect size increased when restricted to BCP ALL, regardless of hyperdiploidy. The two minor allele variants in *IKZF1* also showed significant associations with total ALL (*p* value = 7.8 × 10^−6^ and 8.4 × 10^−6^, respectively) among non-Hispanic Whites, and their effects were similar across disease subgroups. For *CDKN2A*, none of the five studied variants was significantly associated with ALL among non-Hispanic Whites. However, one *CDKN2A* SNP, rs3731217, showed a suggestive association with total ALL [OR 0.71 (0.49–1.03)] that gained strength and reached significance when restricted first to BCP ALL [OR 0.66 (0.44–0.99)] and then to BCP high-hyperdiploid ALL [OR 0.46 (0.22–0.95)].

**Table 2:** Association of candidate SNPs with childhood ALL risk, non-Hispanic Whites, and Hispanics

Gene	SNP	Minor allele	Non-Hispanic Whites					Hispanics				
			MAF^a^ (%)	Total ALL		B-cell	B-cell hyperdiploid	MAF^a^ (%)	Total ALL		B-cell	B-cell hyperdiploid
				Allelic OR^b^ (CI)	P^c^	Allelic OR^b^ (CI)	Allelic OR^b^ (CI)		Allelic OR^b^ (CI)	P^c^	Allelic OR^b^ (CI)	Allelic OR^b^ (CI)
*ARID5B*	rs10994982	A	48	**1.37 (1.08**–**1.73)**	**0.018**	**1.41 (1.10**–**1.80)**	**1.67 (1.14**–**2.47)**	54	**1.53 (1.23**–**1.90)**	**0.0004**	**1.65 (1.31**–**2.07)**	**2.21 (1.52**–**3.21)**
*ARID5B*	rs10740055	C	48	**1.50 (1.18**–**1.90)**	**0.003**	**1.57 (1.23**–**2.02)**	**1.76 (1.19**–**2.61)**	54	**1.61 (1.29**–**2.00)**	**1** × **10** ^−**5**^	**1.72 (1.37**–**2.17)**	**2.43 (1.67**–**3.55)**
*ARID5B*	rs7073837	A	45	**1.43 (1.11**–**1.85)**	**0.009**	**1.59 (1.20**–**2.11)**	**2.30 (1.48**–**3.59)**	52	**1.76 (1.39**–**2.23)**	**4.3** × **10** ^−**7**^	**2.23 (1.68**–**2.94)**	**2.66 (1.75**–**4.03)**
*ARID5B*	rs7089424	G	29	**1.84 (1.43**–**2.37)**	**2.2** × **10** ^−**6**^	**1.98 (1.50**–**2.61)**	**2.71 (1.79**–**4.12)**	39	**1.98 (1.59**–**2.48)**	**1.0** × **10** ^−**9**^	**2.20 (1.72**–**2.81)**	**3.22 (2.18**–**4.73)**
*ARID5B*	rs10821936	C	31	**1.79 (1.40**–**2.28)**	**4.8** × **10** ^−**6**^	**1.83 (1.42**–**2.36)**	**2.43 (1.64**–**3.61)**	41	**1.99 (1.61**–**2.47)**	**1.2** × **10** ^−**9**^	**2.15 (1.70**–**2.71)**	**3.08 (2.11**–**4.48)**
*CEBPE*	rs2239633	C	51	**1.44 (1.13**–**1.82)**	**0.005**	**1.83 (1.42**–**2.36)**	**1.88 (1.25**–**2.82)**	61	1.24 (0.99–1.55)	0.0671	**1.26 (1.00**–**1.58)**	**1.81 (1.24**–**2.66)**
*IKZF1*	rs11978267	G	27	**1.81 (1.41**–**2.32)**	**7.8** × **10** ^−**6**^	**1.78 (1.37**–**2.30)**	**1.76 (1.18**–**2.62)**	27	1.20 (0.95–1.51)	0.1483	1.18 (0.93–1.50)	1.21 (0.84–1.73)
*IKZF1*	rs4132601	G	27	**1.80 (1.41**–**2.31)**	**8.4** × **10** ^−6^	**1.78 (1.37**–**2.30)**	**1.75 (1.18**–**2.61)**	27	1.22 (0.97–1.54)	0.08	1.21 (0.95–1.54)	1.27 (0.88–1.82)
*CDKN2A*	rs3731217	G	14	0.71 (0.49–1.03)	0.079	**0.66 (0.44**–**0.99)**	**0.46 (0.22**–**0.95)**	9	0.74 (0.50–1.11)	0.162	0.70 (0.46–1.07)	**0.38 (0.16**–**0.89)**
*CDKN2A*	rs3218018	C	9	0.99 (0.67–1.48)	0.966	0.99 (0.65–1.53)	1.24 (0.66–2.32)	6	**1.78 (1.19**–**2.66)**	**0.0145**	**1.77 (1.18**–**2.66)**	**2.37 (1.40**–**4.00)**
*CDKN2A*	rs2811712	G	10	0.96 (0.66–1.41)	0.921	1.05 (0.70–1.57)	1.23 (0.33–2.25)	8	**1.56 (1.09**–**2.24)**	**0.0315**	**1.53 (1.06**–**2.22)**	**1.91 (1.17**–**3.11)**
*CDKN2A*	rs3731239	C	36	1.26 (0.95–1.55)	0.163	**1.32 (1.02**–**1.71)**	**1.55 (1.05**–**2.29)**	32	0.97 (0.74–1.27)	0.7407	0.99 (0.74–1.31)	1.04 (0.68–1.58)
*CDKN2A*	rs4074785	A	10	0.90 (0.59–1.38)	0.623	1.03 (0.66–1.60)	0.92 (0.45–1.88)	29	0.96 (0.76–1.22)	0.633	1.00 (0.78–1.28)	0.96 (0.65–1.41)

*MAF* minor allele frequency, *SNP* single nucleotide polymorphism, *OR* odds ratio, *CI* 95 % confidence interval. Bold typeface indicates results with p < 0.05 and/or odds ratios whose 95% confidence intervals exclude 1

^a^Minor alleles defined a priori to match previous GWAS papers; reported MAFs among controls may exceed 50 % in the present sample

^b^OR adjusted for age and gender

^c^ *p* value from log-additive inheritance model

The effects of the 13 variants with childhood ALL risk among Hispanics are also shown in Table 2. Similar to the effects observed among non-Hispanic Whites, all five *ARID5B* SNPs were significantly associated with the risk of total ALL (*p* values from 0.0004 to 1 × 10^−9^). As before, effect sizes were notably stronger when restricted to BCP high-hyperdiploid ALL, but among Hispanics, this strengthening was evident for all five SNPs (OR for BCP high-hyperdiploid ALL is 1.5 times that of total ALL). The minor alleles of the five *ARID5B* SNPs were more common, and risk estimates were somewhat stronger among Hispanics than among non-Hispanic Whites. Among Hispanics, the single SNP in *CEBPE* was suggestively associated with total ALL (*p* = 0.0671) and showed the same strengthening of the effect for BCP high-hyperdiploid ALL [OR 1.81 (1.24–2.66)] as was observed among non-Hispanic Whites, but not total BCP ALL. For the *IKZF1* SNPs, although the risk estimates among Hispanics were the same direction as those among the non-Hispanic Whites, they were of lower magnitude (ORs 1.20–1.22 vs. 1.80–1.81 among non-Hispanic Whites), and the *p* values were not significant, and these effects did not differ by disease subgroup. Finally, two of the five SNPs in *CDKN2A*, rs3218018 and rs2811712, were significantly associated with childhood ALL among Hispanics (*p* = 0.0145 and 0.0315), and this effect was strongest for BCP high-hyperdiploid ALL. These associations were not observed among non-Hispanic Whites.

## Discussion

In this study we examined the associations of previously identified childhood ALL risk loci in both non-Hispanic Whites and Hispanics, whose recent admixture and elevated incidence rates of childhood ALL warrant special attention in studies of genetic susceptibility. To our knowledge, this is the first study to examine the role of these variants in a Hispanic population. Our results indicate that while some previously identified susceptibility loci, including those in *ARID5B* and *CEBPE*, are consistent across the two studied populations, others, including those in *IKZF1* and *CDKN2A*, may have different effects.

The genes studied here were identified through previous genome-wide interrogation and replication in European White populations. Given the suspected roles of *ARID5B*, *CEBPE*, and *IKZF1* in B-cell development, the biological plausibility of their involvement in ALL etiology is strong. We expected the non-Hispanic White population in our study to closely resemble the European Whites reported in the previous studies [1–4]. In this group, we found results for SNPs in *ARID5B*, *CEBPE*, and *IKZF1* that were remarkably similar to those from the previous studies, including both the direction and magnitude of previously reported effects.

The consistency of results from our non-Hispanic White population with those from European White populations, taken together with the consistency of observed effects of loci in *ARID5B*, and to a lesser extent *CEBPE*, between Hispanic and non-Hispanic White populations in our study, provides compelling support that these loci are indeed involved in risk of childhood ALL, particularly BCP high-hyperdiploid ALL. The higher observed risk estimates for these loci in Hispanics versus non-Hispanic Whites are intriguing. If real, they may be due to the differences in either the genetic structure in the region of these variants or the prevalence of exposures (including environmental/lifestyle exposures) that modify the risks associated with these variants.

While we did not observe statistically significant associations of the two studied *IKZF1* SNPs with childhood ALL susceptibility among Hispanics, the direction of observed effects for the specified allele was similar to those observed among non-Hispanics. In addition, the *p* value for rs4132601 among Hispanics was borderline significant (*p* = 0.08), suggesting that, with a larger sample and therefore greater statistical power, the association could be significant. It is possible that the *IKZF1* associations observed among our non-Hispanic Whites and among the European White populations in the previous studies [1–4] are indirect, due to linkage disequilibrium with functional loci elsewhere. In this case, effects for the studied *IKZF1* loci, and indeed other loci whose observed susceptibility effects differ between populations, might be obscured by differences in linkage patterns due to the recent admixture of the Hispanic population.

Separately, the effects of *CDKN2A*, a cell cycle control gene known to be a tumor suppressor, were not strong enough to reach genome-wide significance in the previous genome-wide studies of European Whites and therefore required a larger independent replication sample for final identification [6]. Among the non-Hispanic Whites in our study, we observed suggestive associations of the same *CDKN2A* variant previously reported among European Whites [6]. However, among Hispanics, two different *CDKN2A* loci were significantly associated with childhood ALL. As noted above, this observed difference between Hispanics and non-Hispanic Whites may be due to either small effects of the *CDKN2A* loci, which would require larger sample sizes for consistent observation, or genetic heterogeneity surrounding the *CDKN2A* locus. In summary, although *CDKN2A* shows some effects, further studies are needed to clarify whether or not *CDKN2A* variants are indeed childhood ALL susceptibility loci.

In this study, our goal was to attempt to replicate the previous findings and determine the effect sizes of previously identified susceptibility loci in both a replication population (non-Hispanic Whites) and a different, higher-risk population (Hispanics). Accordingly, we did not impose control for multiple testing—all reported *p* values are nominal. While application of approaches such as the false discovery rate [18] would undoubtedly render some of the findings reported here nonsignificant, several loci would likely persist, notably those in *ARID5B*, due to their low nominal *p* values.

In our race- and ethnicity-matched study population, we have previously calculated genetic ancestry on the basis of a panel of 96 ancestry informative markers selected to distinguish continental origin among California Hispanics [19]. As part of this work, we found that adjustment for the child’s race and Hispanic ethnicity, as reported by the mother, yields individual SNP risk estimates not substantially different from those derived from models adjusted for estimated genetic ancestry [20]. We observed similar minimal changes in SNP risk estimates after further adjustment for genetic ancestry (data not shown); accordingly, we report the effects of the most parsimonious model, adjusted only for race (a matching factor). Based on these results, it is unlikely that the observed risk estimates, or the observed susceptibility differences across populations, are due solely to latent population stratification or cryptic relatedness.

In conclusion, we found that previously identified childhood ALL susceptibility loci in *ARID5B* and *CEBPE* show consistent risk effects across both Hispanic and non-Hispanic White populations, providing compelling supportive evidence for susceptibility at these loci. In contrast, *IKZF1* and *CDKN2A* variants displayed varying susceptibility loci between populations. Future studies with larger sample sizes should examine the effects of these variants in other subgroups of ALL and examine whether these effects vary by other environmental (immunological) factors/lifestyle factors.
